# Inferring the timing of individual mobility decisions from accommodation reservation data during the COVID-19 outbreak

**DOI:** 10.1098/rsos.250554

**Published:** 2025-07-30

**Authors:** Koichi Ito, Shunsuke Kanemitsu, Ryusuke Kimura, Ryosuke Omori

**Affiliations:** ^1^ Faculty of Science and Engineering, Doshisha University, Kyotanabe, Kyoto 610-0394, Japan; ^2^ Data Solution Unit 2 (Marriage & Family/Automobile Business/Travel), Data Management & Planning Office, Product Development Management Office, Recruit Co., Ltd, Tokyo 100-6640, Japan; ^3^ SaaS Data Solution Unit, Data Management & Planning Office, Product Development Management Office, Recruit Co., Ltd, Tokyo, 100-6640, Japan; ^4^ Division of Bioinformatics, International Institute for Zoonosis Control, Hokkaido University, Sapporo, Hokkaido 001-0020, Japan

**Keywords:** COVID-19, risk reduction behaviours, data interpretations, outbreaks, human mobility, population dynamics, epidemiology

## Abstract

Understanding the changes in human mobility in response to outbreaks is important for controlling emerging infectious disease outbreaks. This requires an understanding of the mechanism of human behavioural response as well as the timing of decisions for future mobility. However, most human mobility data only record the executed mobility that results from decision-making, and not the timing of decisions. In this study, we used accommodation reservation data to extract the decision-making process in response to the changing epidemic situation and compared it with data on executed mobility, ‘stay time’ in workplaces and stay time in places other than home or workplaces to clarify when people decide on their mobility. We confirmed that the decision-making process estimated from accommodation reservation data can accurately predict human mobility. The decision-making process estimated from accommodation reservation data was more strongly associated with stay time in places other than home or workplaces than stay time in workplaces. Furthermore, the comparison between the estimated decision-making process and mobility data quantitatively revealed that mobility was the result of integrating two types of decisions made in recent weeks (within two and five weeks for mobility to workplaces and places other than home or workplaces, respectively) and previous weeks.

## 1. Introduction

The COVID-19 outbreak reinforced the necessity of predicting the epidemic dynamics of emerging infectious diseases. Several methods for predicting epidemic dynamics can be considered, one of which is mathematical modelling of epidemic dynamics such as the susceptible, infectious or recovered (SIR) model [1]. The SIR model describes the transmission of pathogens through contact between infected and susceptible hosts. The simplest SIR model assumes a homogeneous host-contact process, which implies the same contact probability between hosts. To capture the variability of contact probabilities between hosts, the SIR model was expanded to follow real data from the contact processes, such as the spatial-dependent contact [2] and age-dependent contact processes [3].

**Figure 1.**
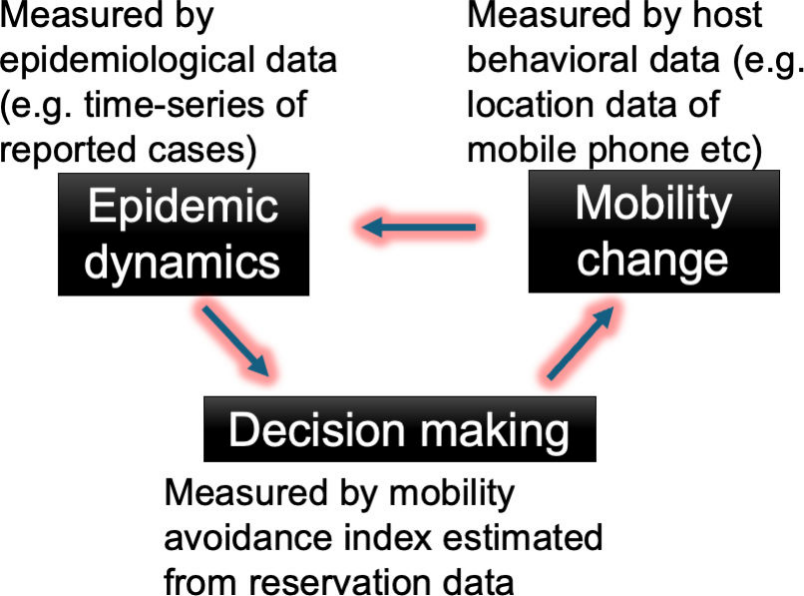
Mechanism of host mobility changes in response to epidemics. To predict the future course of epidemic dynamics from previous data, the impacts of (i) epidemic dynamics on decision-making for mobility change, (ii) decision-making on mobility change, and (iii) mobility change on epidemic dynamics are considered.

Another factor of heterogeneity in contact probabilities is host mobility changes in response to an epidemic. Hosts change their mobility in response to an epidemic situation for several reasons such as non-pharmaceutical interventions [4–7] and voluntary mobility changes [8–11]. Furthermore, the intensity of such responses changes with time owing to adaptation [12,13], tiredness [14,15] or habituation [16,17]. The host’s mobility changes alter epidemic dynamics, and the epidemic dynamics change the host’s mobility; therefore, simultaneous modelling of epidemic dynamics and host mobility changes is required to predict the future course of epidemic dynamics [18]. For the simultaneous modelling of epidemic dynamics and host mobility changes, quantification of the associations between them is required (figure 1). The use of host behavioural data, such as location data from mobile phones, Short Message Service (SMS), or integrated circuit (IC) cards on public transportation, enables the estimation of the impact of host mobility changes on epidemic dynamics [19–29]. Indeed, the use of host mobility changes improves the prediction of epidemic dynamics; for example, time-series data of reported cases [30,31]. However, understanding the impact of epidemic dynamics on changes in host mobility is difficult. Because people often schedule their future behaviours in advance, the observed mobility data can be considered an accumulation of past decision-making. The timing of the decision for the observed mobility cannot be determined from behavioural data; thus, the epidemic situation that triggered the mobility changes cannot be understood from only a comparison of epidemiological and host behavioural data (figure 1). To understand people’s mobility changes against the epidemic dynamics, measurement of the decision-making process is necessary. However, mobility data record only the results of decision-making and not the timing of decision-making.

Ito *et al.* proposed a solution to this problem using accommodation reservation data. The reservation dataset consisted of the reservation timing, scheduled time and cancellation timing if the reservation was cancelled [32]. Making a new reservation was considered a decision-making process that increases contact with others, and cancellation of reservations can be considered a decision-making process that reduces contact. The reservation data record the timing of such decision-making. Ito *et al.* proposed and measured the mobility avoidance index in response to the COVID-19 outbreak using an accommodation reservation dataset [32]. Omori *et al.* estimated the impact of epidemic dynamics (time-series data of reported COVID-19 cases) on the mobility avoidance index and found that mobility avoidance changed in proportion to the logarithm of the number of reported cases [33].

Previous studies have shown that human decision-making processes in response to epidemic dynamics can be measured using accommodation reservation data [32,33]. Although mobility is determined by the accumulation of decision-making over the previous days, the final update in decision-making determines actual mobility. The timing of the final update is important for understanding the impact of an epidemic on host mobility. The timing of the final update can be obtained by comparing the measured decision-making process from the accommodation reservation data with mobility data as the outcome of the decision-making process, such as the location data of mobile phones. However, accommodation reservations are accompanied by a limited part of people’s mobility; thus, it has not been guaranteed that the measured decision-making represents those for more general mobilities, such as commuting, shopping, eating out or one-day leisure trips. In this study, the predictability of general mobility, which was recorded as the location data of mobile phones, was assessed using the mobility avoidance index estimated from accommodation reservation data. Moreover, the timing of decision-making may have a strong impact on future mobility. Therefore, the aim of this study was to understand the timing of decisions for future mobility during an emerging infectious disease outbreak and the impact of an epidemic on host mobility changes.

## 2. Methods

### 2.1. Data

For the evaluation of the people’s general mobility, the ‘Konzatsu-Tokei (r)’ Data provided by ZENRIN DataCom Co. Ltd. is used. This is people flows data collected by individual location data sent from mobile phone through applications provided by NTT DOCOMO, INC. These data were processed to protect privacy, and they do not include the information to specify individual. General mobility amount is quantified by the ‘stay time’, which is an indicator contained in the Konzatsu-Tokei (r) Data, representing the people’s stay amount in a focal location, with one person staying for 15 min being counted as one. Each stay is labelled as ‘home’, ‘workplace’ or ‘other’ based on past location data of the focal person (i.e. the most and the second most frequently stayed places are estimated as ‘home’ and ‘workplace’, respectively). Additionally, based on the estimated ‘home’ location, each stay is also classified by the place of visitor’s residence. Based on these, the weekly total ‘stay time’ in each city in four prefectures (Miyagi, Aichi, Osaka, Fukuoka) from 6 August 2018 to 4 December 2022 with disaggregated by location type (workplace and other) and by visitor’s place of residence was calculated and anonymized before it was provided to us. For the detail of the Konzatsu-Tokei (r) Data, see Data Accessibility.

To estimate the mobility avoidance index [32], the travel agency’s online accommodation reservation data (https://www.jalan.net/) were used, which excluded personally identifiable information. To compare human mobility before and after COVID-19, we used reservation and cancellation data from 28 December 2015 to 19 February 2023, for accommodations located in Miyagi, Aichi, Osaka and Fukuoka prefectures. The number of reported COVID-19 cases was obtained from the website of the Ministry of Health, Labour and Welfare of Japan [34].

### 2.2. Calculation of the mobility avoidance index from reservation data

Using the method proposed by Ito *et al.* [32], we estimated the mobility avoidance index, which is measured by the influence of humans’ decisions to make a new accommodation reservation or cancel an existing reservation. Using the avoidance index at week *t* for mobility *x* days ahead *λ_t,x_*, the reduction rate for new reservations for *x* days ahead *R_t, x_* was modelled as


(2.1) $$R_{t,x}=\frac{1}{1+exp\left(a\left(logit\left(\lambda_{t,x}\right)-b\right)\right)}.$$

The increased rate of cancellations of the existing mobility for *x* days ahead *C_t,x_* was modelled as follows:


(2.2) $$C_{t,x}=\frac{1}{1+\;exp\left(c\left(logit\left(\lambda_{t,x}\right)-d\right)\right)},$$

where


(2.3) $$logit\left(\lambda \right)=\;ln\left(\frac{\lambda }{1-\lambda }\right),$$

and *λ_t,x_* = 0 refers to a score which is equal to the average mobility avoidance index before the emergence of COVID-19, and 1 means that no new mobility is planned and all planned mobilities are cancelled. Using equations (2.1) and (2.2), the expected number of reservations for stay *x* days ahead, at time *t*, which is reduced by *λ_t,x_* is


(2.4) $$R_{t,x}^{*}={\overset{-}{R}}_{x}\left(1-R_{t,x}\right),$$

where ${\overset{-}{R}}_{x}$ is the average number of new reservations for stay *x* days before the emergence of COVID-19. Similarly, the cancellation probability of the existing reservations per day is represented as


(2.5) $$C_{t,x,y}^{*}={\overset{-}{C}}_{x,y}+\left(1-{\overset{-}{C}}_{x,y}\right)C_{t,x},$$

where ${\overset{-}{C}}_{x,y}$ is the average cancellation probability of the reservation, which is reserved y days ahead of the stay and cancelled *x* days ahead of the stay prior to the emergence of COVID-19. As shown in Ito *et al.* [32], we estimated $\lambda_{t,x}$ by fitting $R_{t,x}^{*}$ and $C_{t,x,y}^{*}$ to the accommodation reservation data using maximum likelihood estimation (electronic supplementary material, S1). The maximum likelihood estimate of $\lambda_{t,x}$, $\lambda_{t,x}^{*}$, was normalized by the average $\lambda_{t,x}$ before the emergence of COVID-19, ${\overset{-}{\lambda }}_{t,x}$, such that, 0 is equal to the average $\lambda_{t,x}$ before the emergence of COVID-19, and 1 is the theoretical maximum value of $\lambda_{t,x}$ as


(2.6) $${\hat{\lambda }}_{t,x}=\frac{\lambda_{t,x}^{*}\;-{\overset{-}{\lambda }}_{x}}{1-{\overset{-}{\lambda }}_{x}}.$$

The normalized $\lambda_{t,x}$, ${\hat{\lambda }}_{t,x}$ was smoothed by the locally weighted smoothing method along both week *t* and x days direction.

### 2.3. Prediction of general mobility using the mobility avoidance index

Humans often cancel travel plans or attend events prior to the date of mobility. Therefore, the general mobilities observed in a focal week could be reduced not only by the travel avoidance index during the focal week but also by the travel avoidance index in the past for that week. General human mobility is determined by the accumulation of the travel avoidance index over the past few weeks. We simply assumed that the general mobility amount in week *t* was approximately reduced by the weighted summation of the past travel-avoidance index for the focal week,


(2.7) $$M_{K,t}^{*}=M_{0}\left\{1-\sum_{\tau =0\ldots 53}F_{K}\left(\tau \right)\lambda_{t-\tau ,\tau }\right\},$$

where $M_{0}$ is the baseline general mobility amount, $F_{K}(\tau )$ is the impact of the past decisions indicated by travel avoidance index on the reduction of general mobility. For $F_{K}\left(\tau \right)$, the sum of exponential functions is assumed, i.e.


(2.8) $$F_{K}\left(\tau \right)=\sum_{i=1\ldots K}a_{i}\;\exp\left(b_{i}\tau \right),$$

where *K* determines the number of exponential functions.

We assumed that the observed general mobility during the focal week followed a Poisson distribution, whose expected occurrence number is $M_{K,t}^{*}$. Accordingly, the likelihood function of $M_{K,t}^{*}$ when the number of exponential functions in $F_{K}\left(\tau \right)$ is *K* is as follows:


(2.9) $$L_{K}\left(a_{1\ldots K},b_{1\ldots K},M_{0}\right)=\prod_{t}pmf\left(poisson\left(M_{K,t}^{*}\right),M_{t,Data}\right),$$

where $M_{t,Data}$ is the observed stay time on week *t*, obtained from the Konzatsu-Tokei (r) Data.

### 2.4. Estimation, cross-validation and confidence interval

The coefficients of the model for the impact of past decisions, $a_{1\ldots K}$, $b_{1\ldots K}$, and *M*_0_ in equation (2.8), maximizing the likelihood *L_K_* under the given subscripts *K* and $M_{t,Data}$ were estimated using the Nelder–Mead method: to compare the performances of the models with different subscripts *K*, we applied leave-one-out cross-validation. The mobility avoidance index and general mobility data were partitioned by the week defined in ISO 8601 (partitioned into 213 sub-datasets). We calculated the coefficients maximizing the likelihood *L_K_* estimated using all sub-datasets other than the *i*th week sub-dataset, as well as the estimated coefficients; accordingly, the likelihood of the *i*th week sub-dataset and its Bayesian information criterion (BIC) [35] were calculated. The performance of each model was evaluated using the mean BIC value.

The confidence intervals (CIs) of the model coefficients and estimations were computed using the bootstrap method. The mobility avoidance index and general mobility data were partitioned again into 213 weekly sub-datasets. The bootstrap sample was obtained by random sampling with the replacement of the 213 sub-datasets, and the model coefficients and estimation of the model were obtained by maximum likelihood estimation. By repeating this process 100 000 times, the CIs of the model coefficients and the estimated general mobility of the model were computed. The goodness of the model estimation was evaluated using the coefficient of determination, *R*^2^.

The impact of the past travel avoidance index on general mobility may vary with calendar time. Omori *et al.* reported that, with respect to the relationship between the travel avoidance index and the weekly number of reported COVID-19 cases, the COVID-19 epidemic waves in Japan can be grouped into three clusters: the first cluster (the first wave; from 13 January to 14 June 2020), middle cluster (the second to fifth epidemic waves; from 15 June 2020 to 28 November 2021) and late cluster (the sixth and latter waves; from 29 November 2021 to 4 December 2022) [33].

## 3. Results

### 3.1. Estimation of general mobility changes from accommodation reservation data

**Figure 2.**
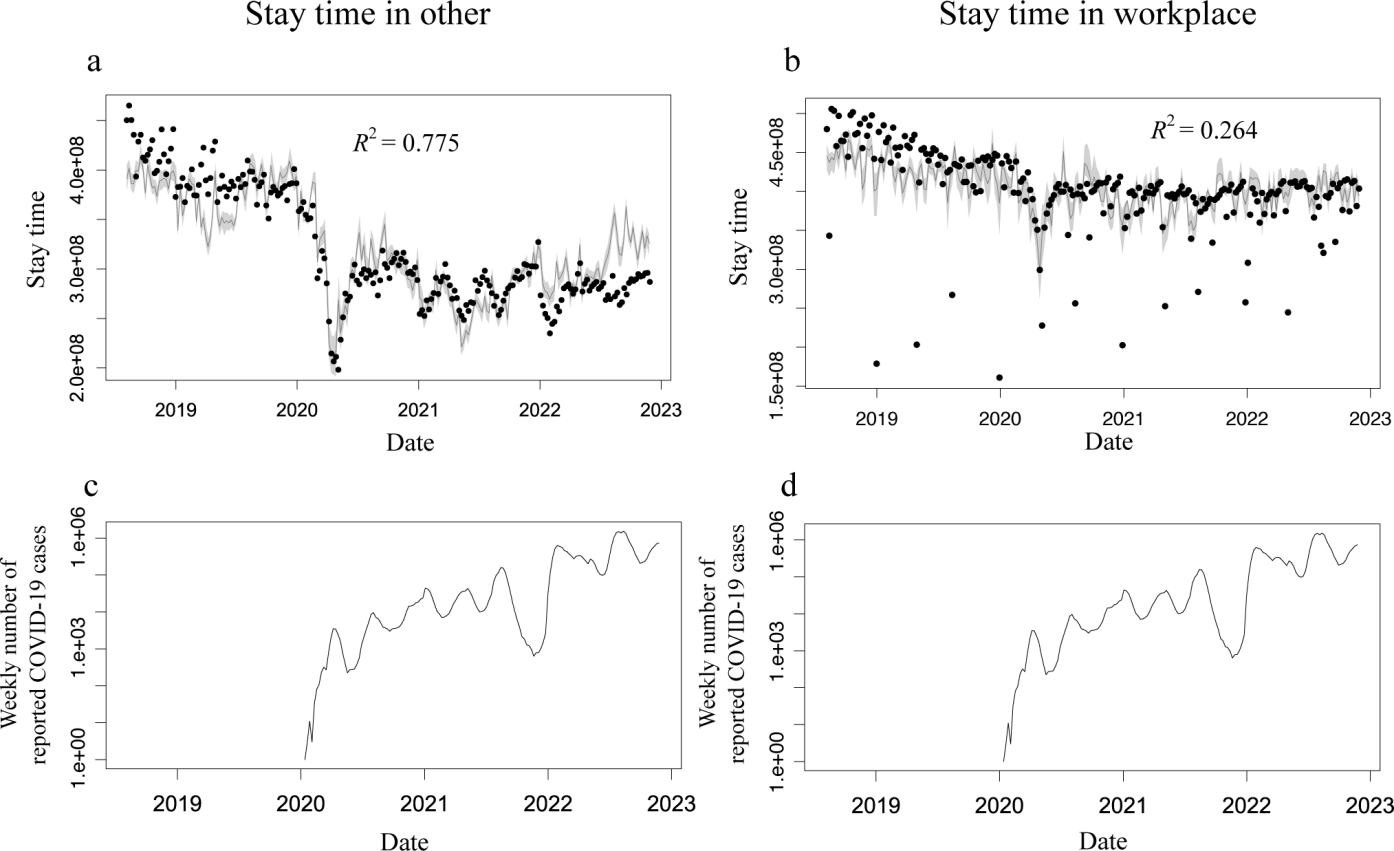
Comparison of the estimated and observed stay time. Upper panels show the stay time in other (a) and workplace (b). The data (black dots) and the estimation by the best model (black curve) with 95% confidential interval computed using the bootstrap method (grey band). Lower panels show the weekly number of reported COVID-19 cases (logarithmic in Y-axis). The results are calculated using ‘Konzatsu-Tokei (r)’ Data.

Figure 2 shows a comparison between the estimated ‘stay time,’ observed stay time and COVID-19 epidemic status. Although both mobility in other places and that in workplaces (black dots in the upper panels in figure 2) seem to change in response to the number of newly reported COVID-19 cases (lower panels in figure 2), the reduction in mobility in other places was larger than that in the workplace. The stay times estimated in other locations using the best model followed the actual observed data well (coefficient of determination *R*^2^ = 0.775, figure 2a). However, the estimation accuracy of the stay time at the workplace location was poor (*R*^2^ = 0.264, figure 2b). To remove the effect of the long holiday period, we generated a sub-dataset by excluding major holiday weeks (the week including 1 January, 4 May or 13 August, on which the stay time in the workplace was less than 80% of the average during 2018 and 2019), but the improvement in the estimation accuracy was small (*R*^2^ = 0.480, electronic supplementary material, figure S1).

**Figure 3.**
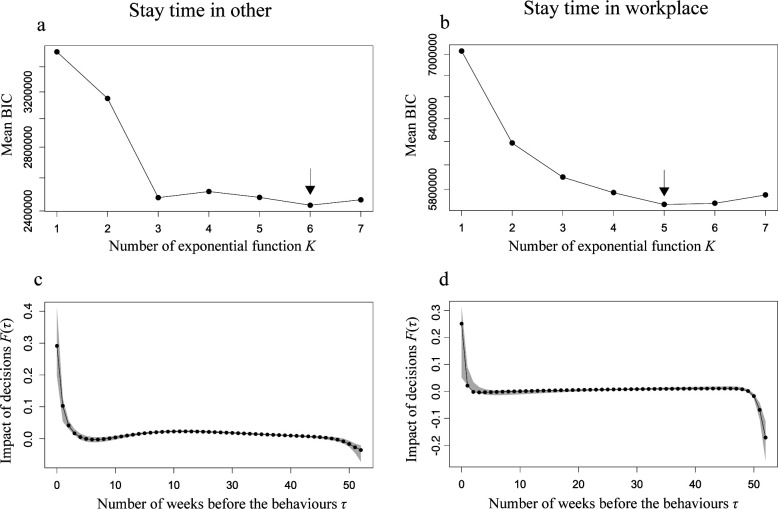
Performance of the models for estimation of general mobility. Upper panels show the mean Bayesian information criterion (BIC) in the leave-one-out cross-validation for the model of the stay time in other (a) and workplace (b). Horizontal axis shows the number of exponential function *K*. Directing arrow indicates the best exponential function *K*, where the mean BIC is minimum. Lower panels show the impact of the past decisions indicated by travel avoidance index F(τ) with the best exponential function for the model of the stay time in other (c) and workplace (d). Grey band indicates the 95% confidential interval computed using the bootstrap method. The results are calculated using ‘Konzatsu-Tokei (r)’ Data.

Figure 3a,b shows the mean BIC of the model $M_{K,t}^{*}$ with the variation in the number of exponential function *K* (horizontal axis) for the stay time in the ‘other’ and ‘workplace’ locations, respectively. The best exponential functions *K* for other places and workplaces were six and five, respectively. Figure 3c,d shows the impact of the past decisions indicated by travel avoidance index F(τ) with the best exponential function number. The impact of past decisions decayed within approximately five weeks in the model for others and within two weeks in the model for the workplace. Interestingly, the impacts of the decisions made more than a few months ago were non-zero values; that is, the positive and negative impacts were estimated at approximately 10−40 weeks and more than 50 weeks for both models.

### 3.2. Effect of calendar time, geographical location and mobility distance

**Figure 4.**
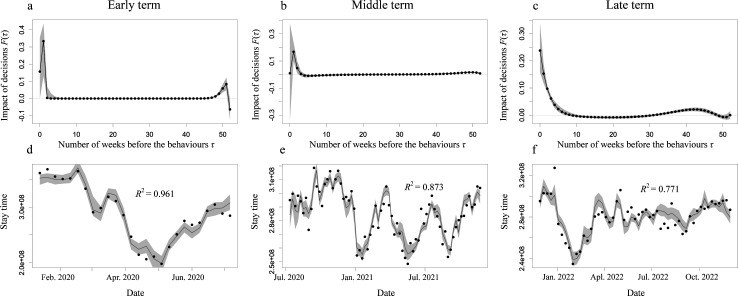
Effect of the calendar time on the estimation of stay time in other locations. Upper panels show the impact of the past decisions indicated by travel avoidance index F(τ) with the best exponential function for the model of the stay time in other locations during the early cluster (a), middle cluster (b) late cluster (c). Grey band indicates the 95% confidential interval computed using the bootstrap method. The lower panels show the stay time in other locations during the early cluster (d), middle cluster (e) and late cluster (f). In each panel, the data (black dots) and the estimation by the best model (black curve) with 95% CIs computed using the bootstrap method (grey band). The results are calculated using ‘Konzatsu-Tokei (r)’ Data.

Owing to the low accuracy in the estimate of stay time in the workplace in the above analyses, we focused only on the estimation of stay time in other locations. Electronic supplementary material, figure S2 shows the best exponential function *K* selected based on the mean BIC in the leave-one-out cross-validation following division of the dataset into three calendar clusters [33]. Figure 4a–c shows the estimated impact of past decisions on each cluster in the best model. Decay in past decisions tends to be slower in later clusters; that is, decisions on the focal or previous week had the greatest impact in the early cluster, while decisions in the past nine weeks still had an impact in the late cluster. The estimation accuracy (figure 4d–f) was highest in the early cluster and decreased in the later clusters.

The accommodation reservation dataset was divided into four sub-datasets corresponding to each prefecture (Miyagi, Aichi, Osaka and Fukuoka), and the mobility avoidance index in each prefecture was estimated. The general mobility dataset was also divided into four sub-datasets, and the best parameters of the mode of stay time in each prefecture by the mobility avoidance index in each prefecture were estimated. However, the shape of the impact of the past decisions $F_{K}(\tau )$ or the estimation accuracy was not significantly different (electronic supplementary material, figure S3).

**Figure 5.**
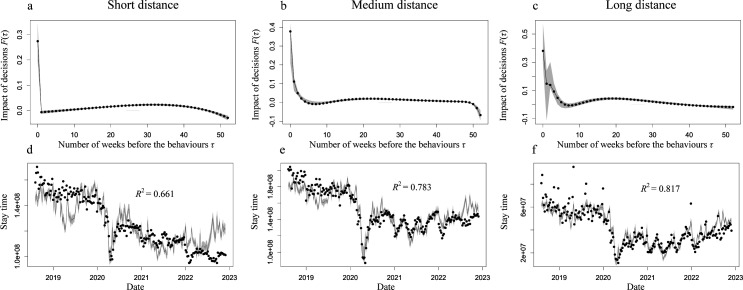
Effect of the movement distance on the estimation of stay time in other locations. Upper panels show the impact of the past decisions indicated by travel avoidance index F(τ) with the best exponential function for the model of the stay time in other locations for short distances (a), medium distances (b) and long distances (c). Grey band indicates the 95% confidential interval computed using the bootstrap method. The lower panels show the stay time in other locations for short distances (d), medium distances (e) and long distances (f). In each panel, the data (black dots) and the estimation by the best model (black curve) with 95% CI are computed using the bootstrap method (grey band). The results are calculated using ‘Konzatsu-Tokei (r)’ Data.

We categorized the mobility distance into three scenario datasets: (i) the stayed place and the place of the visitor’s residence were located within the same city (short distance), (ii) the stayed place and the place of visitor’s residence were not located in the same city, but located within the same prefecture (middle distance), and (iii) the stayed place and the place of visitor’s residence were not located in the same prefecture (long distance). Subsequently, we applied the estimations for each dataset. Electronic supplementary material, figure S4 shows the best exponential functions *K*, selected based on the mean BIC in the leave-one-out cross-validation. Figure 5a–c shows the estimated impact of past decisions on each term in the best model. The decay of past decisions tended to be slower when the mobility distance was longer; that is, at short distances, the decisions from the focal week had a high impact, whereas decisions from the past seven weeks still had an impact over long distances. The estimation accuracy was the highest for long mobility distances (figure 5d–f) and decreased with shorter distances.

## 4. Discussion

In this study, we assessed the usability of the mobility avoidance index estimated using accommodation reservation data. Precisely, the predictability of the stay time in the ‘workplace’ and in the ‘other’ places (i.e. other than home and workplace) by mobility avoidance index estimated from the accommodation reservation data was assessed. The stay time in other places and workplaces was accurately predicted from the mobility avoidance index estimated from accommodation reservation data; however, stay time in the workplace could not be predicted. In addition, recent decision-making had a strong impact on mobility. Decisions made within five and two weeks before mobility were important for mobility to other places and the workplace, respectively.

This study extends earlier work on inferring behavioural responses to epidemics using accommodation reservation data. The mathematical modelling framework proposed in Ito *et al.* [32] focused on how reservation data could be used to extract behavioural signals, introducing the concept of a mobility avoidance index. Omori *et al.* [33] applied this framework to the data to analyse how the strength of travel avoidance changed in response to the evolving COVID-19 epidemic situation by comparing the mobility avoidance index and the reported number of COVID-19 cases. The present study builds on these foundations by shifting the focus from the strength of mobility avoidance to the timing of decision-making. By aligning the mobility avoidance index with realized mobility data from mobile phone records, we identify when people make decisions that lead to actual changes in mobility behaviour, and how such decisions vary across mobility types.

Mobility avoidance index estimated from accommodation reservation data can predict the mobility to the other places accurately; however, it cannot predict the mobility to the workplace. Mobility to other places changed in response to the COVID-19 pandemic status—that is, the number of reported cases—but mobility to the workplace was not sensitive to the pandemic status. Mobility changes in response to the epidemic occurred only in mobility to other places, suggesting that the general trend in mobility changes can be captured by the mobility avoidance index estimated from accommodation reservation data.

**Figure 6.**
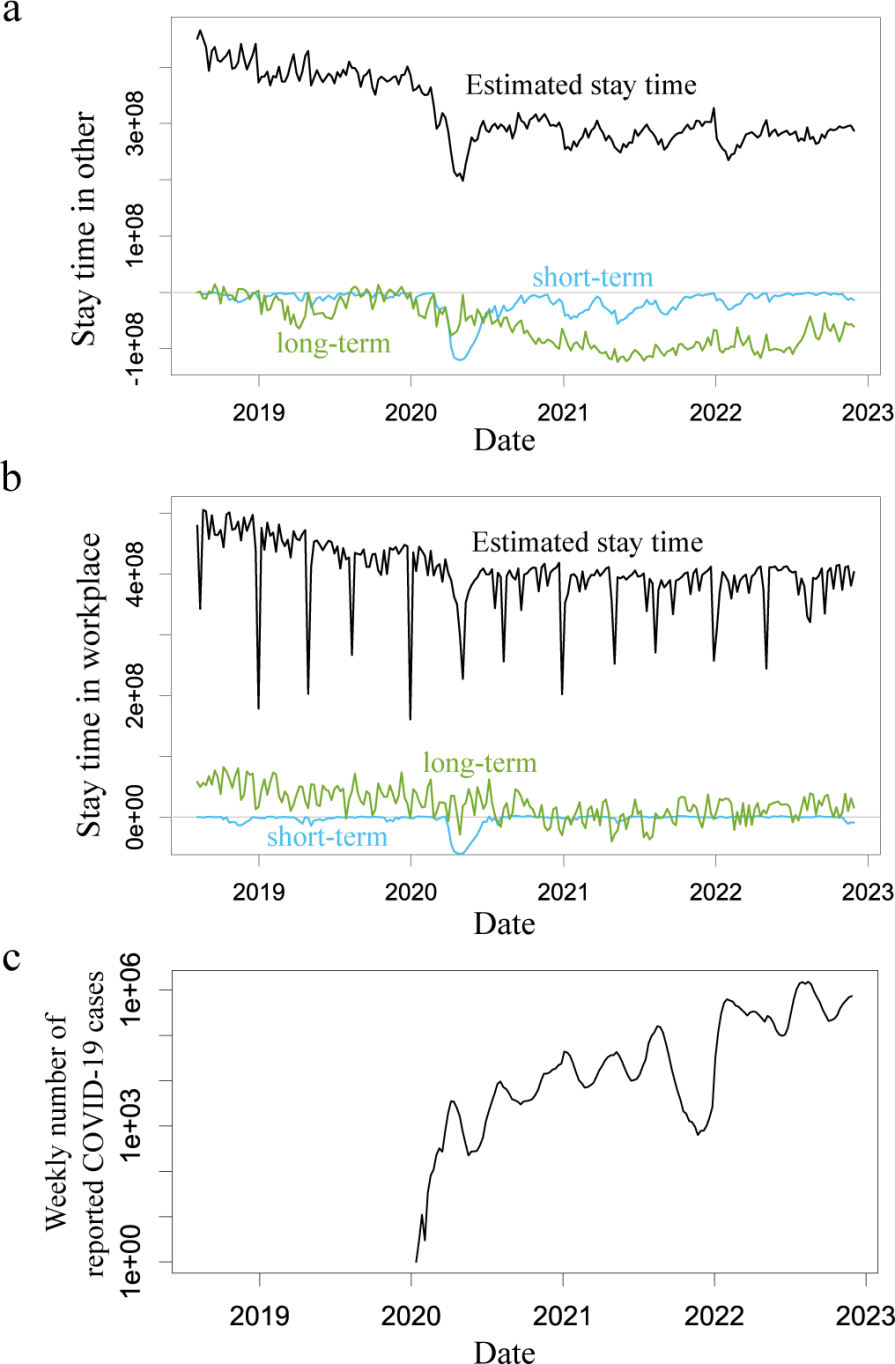
Contribution of the decision-making in recent weeks and prior weeks. Panels a and b show the estimated stay time (black curve), reduction of the mobility due to a decision made within 12 weeks ($\sum_{\tau =0\ldots 12}F_{K}(\tau )\lambda_{t-\tau ,\tau }$; short term, cyan curve) and that made more than 13 weeks ago ($\sum_{\tau =13\ldots 53}F_{K}(\tau )\lambda_{t-\tau ,\tau }$; long term, green curve) in other places (a) and workplace (b), respectively. Panel c shows the weekly number of reported COVID-19 cases (logarithmic in Y-axis). The results are calculated using ‘Konzatsu-Tokei (r)’ Data.

The distributions of the impact of decisions per week are bimodal and can be classified by the decisions made in recent weeks and previous weeks. Recent decisions have been important determinants of mobility to other places. The decision for future mobility can always be updated and it is clear that the most recent decision has the strongest impact on mobility. Indeed, recent decisions contributed to the change in mobility on a small time scale in response to the momentarily changing COVID-19 epidemic situation (cyan curves in figure 6a). Interestingly, regarding the stay time at the workplace, the decisions made in recent weeks had minimal influence compared with those made long ago (the cyan curve is mostly flat compared with the green curve in figure 6b), suggesting that the movement to the workplace can hardly be explained by the epidemic situation. Regarding decisions made in recent weeks, decisions made within three or four weeks still had a high impact on mobility to other places, whereas decisions made within only one to two weeks had a sufficiently high impact on mobility to the workplace (figure 2). This suggests a tendency to take a longer time for scheduling and greater flexibility of mobility to other places compared with workplaces.

However, the strength of the contribution of decisions made long ago to mobility changes varied over a large time scale compared with that of recent decisions. These trends in mobility changes, in terms of sensitivity to the epidemic situation, originate from the trend in the mobility avoidance index (green curves in figure 6a,b). This long-term trend may be caused by the qualitative changes in mobility after the emergence of COVID-19; that is, people tended to reduce mobility frequency itself owing to the spread of the use of remote work or mobile order systems. Indeed, mobility to both other places and workplaces had not returned to the level before the emergence of COVID-19 even in 2023 in Japan (figure 2a,b), and such a constant reduction after the emergence of COVID-19 was realized by the change of decision-making in previous weeks in the estimated model for both other places and workplaces (reduction of green curves realizes the constant reduction of estimated mobility in figure 6a,b). For both mobility to other places and workplaces, the estimated impact of decisions made more than 50 weeks prior is counterintuitive; the oldest decision for mobility negatively affects actual mobility. For example, people who decided upon no mobility avoidance (measured as a low mobility avoidance index) largely suppressed their future mobility. This estimate is derived from the nature of the data on mobility before the emergence of COVID-19. The trend unrelated to the pandemic and the weak impact of COVID-19 on mobility induced an apparent negative correlation between the mobility avoidance index and mobility.

The predictability of mobility changes in response to changes in the epidemic situation was assessed with varied (i) calendar times, (ii) geographical locations, and (iii) mobility distances. With respect to calendar time, we classified the COVID-19 epidemic into three clusters of epidemic waves, as reported by Omori *et al.* [33]: the first wave, the second–fifth waves and the other waves. Comparing the three clusters of epidemic waves, mobility during the early epidemic waves was determined by more recent decisions [33] than during later waves. During the early epidemic waves, the movement to even non-work places required a short scheduling time and had less flexibility of mobility compared with the later waves. This suggests that people restricted their movements to mandatory movements only during the early epidemic waves, but eased the restriction of mobility as a result of habituation to COVID-19 in later waves. Furthermore, the accuracy of the estimation decreased in the later waves, which can be explained by habituation to COVID-19. During the first wave, mobility was mostly determined by the pandemic situation; however, in the latter waves, factors other than the pandemic influenced mobility decisions. In terms of geographical location, no clear difference in the predictability of mobility among the four preferences was observed, suggesting the robustness of predictability with respect to geographical location in Japan. It was easier to predict longer-distance mobility using the mobility avoidance index. The decision for longer-distance mobility was more sensitive to the pandemic situation; thus, it could be predicted more easily.

The mobility avoidance index estimated from accommodation reservation data reflects general mobility changes, regardless of accommodation use. By combining our mathematical model describing the relationship between mobility and the mobility avoidance index (equation (2.7)) and the relationship between the mobility avoidance index and the epidemic situation [33], mobility changes can be predicted from the epidemic situation. Indeed, the actual mobility fluctuated at a low level; although the number of COVID-19 cases increased, the relationship between mobility and epidemics is not simple, such that an increase/decrease in the number of cases decreases/increases mobility. Furthermore, a comparison between the mobility avoidance index and mobility data revealed that mobility was the result of integrating two types of decisions made in recent weeks and those made long ago. This cannot be understood from a comparison between the number of cases and general mobility data.

This study had some limitations. First, this study utilized only data from Japan, and data derived from multiple geographically distant prefectures were used and compared to confirm geographical bias. Consequently, no significant differences within Japan were observed. Nevertheless, the outcomes may differ across countries, and our findings thus require validation using datasets from multiple countries. While this study focused on validating the inferred decision-making process using mobility data derived from mobile phone global positioning system (GPS) records, future research may benefit from comparisons with other sources of realized mobility behaviour, such as Google COVID-19 Community Mobility Reports [36]. These datasets provide complementary perspectives on population-level movement and may help further evaluate the generalizability of our findings across different contexts. Incorporating alternative mobility data into the modelling framework would also allow for a more comprehensive understanding of how various mobility avoidance indices relate to different aspects of human mobility. As such, expanding this analysis to include multiple mobility datasets represents a promising direction for future work. Second, the mobility avoidance index measured from accommodation reservation data did not predict the stay time at workplaces. The development of a statistic that captures decision-making for mobility to workplaces (e.g. a statistic using the history of temporal changes in work schedule) is necessary for better prediction.

In conclusion, the timing of decisions for the majority of mobility during the COVID-19 outbreak was within two and five weeks for mobility to workplaces and places other than home or workplaces, respectively. Our findings have brought us a step closer to the simultaneous prediction of epidemic and mobility changes. They suggest that actual mobility behaviour is influenced by epidemic information accumulated over multiple weeks. Incorporating such temporal lags in behavioural responses into compartmental models such as the SIR model could improve their capacity to represent behavioural mechanisms and enhance the fidelity of epidemic trajectory forecasts. Future research should explore the integration of these lagged decision-making processes into transmission models to better capture the dynamic interplay between epidemic trends and human mobility behaviour.

## Data Availability

The data of accommodation reservation that support the findings of this study are available from Recruit Co., Ltd. (https://recruit-holdings.com/en/), but restrictions apply to the availability of these data, which were used under the license for the current study and are not publicly available. However, the data are available from the authors upon reasonable request and with permission from Recruit Co., Ltd. ‘Konzatsu-Tokei (r)’ is data processed comprehensively and statistically by NTT DOCOMO from cell phone location information sent by consenting users of NTT DOCOMO applications. The location information is GPS data (latitude and longitude information) measured every 5 min at a minimum and does not contain personally identifiable information. ‘Konzatsu-Tokei (r)’ Data is provided by ZENRIN DataCom Co. Ltd. (https://www.zenrin-datacom.net/en/index.html). Restrictions apply to the availability of these data, which were used under the license for the current study and are not publicly available. Data are available with the permission of ZENRIN DataCom Co. Ltd. Supplementary material is available online [37].
